# Evaluating the influence of feedback on motor skill learning and motor performance for children with developmental coordination disorder: a systematic review

**DOI:** 10.3389/fped.2024.1327445

**Published:** 2024-04-19

**Authors:** Ellana Welsby, Brenton Hordacre, David Hobbs, Joanne Bouckley, Emily Ward, Susan Hillier

**Affiliations:** ^1^ Innovation, Implementation, and Clinical Translation (IIMACT) in Health, Allied Health & Human Performance, University of South Australia, Adelaide, SA, Australia; ^2^ College of Science and Engineering, Medical Device Research Institute, Flinders University, Adelaide, SA, Australia

**Keywords:** developmental coordination disorder (DCD), motor performance, motor learning, internal feedback, external feedback, augmented feedback, technology

## Abstract

**Introduction:**

Children with developmental coordination disorder (DCD) have difficulties with learning and performing physical tasks. It is well known that task-specific practice is effective in improving motor skills. Additional feedback during practice may function as a quality improvement mechanism and therefore enhance motor skill outcomes.

**Aims:**

To investigate the effect of different forms of feedback on motor learning and motor performance in children with DCD.

**Methods:**

A systematic review was conducted (registration CRD42020175118) to investigate the effectiveness of different types of feedback, compared to other forms of feedback, or no additional feedback, on motor learning and motor performance outcomes in children with DCD. The search was run across six electronic databases (last search January 2024). Two reviewers independently screened studies for inclusion, assessed the quality of included studies, and extracted relevant data. A narrative synthesis was performed and included studies that assessed motor learning and/or performance outcomes following an intervention that delivered a specific form of feedback in comparison to another form of feedback or no specific feedback.

**Results:**

14 articles from 13 trials were included in this review. Feedback was delivered by providing various forms of feedback, including: knowledge of results, focus of attention and augmented feedback delivered via technology. No significant differences were found between different forms of feedback for motor learning or performance outcomes for children with DCD. Interventions that used technology (with augmented feedback) to deliver the intervention were found to be as effective as traditional therapy. All groups who participated in therapy, regardless of the presence or type of feedback received, improved in overall scores on a motor performance outcome assessment.

**Conclusion:**

Despite the clear rationale for using feedback-oriented interventions for children with DCD, there is surprisingly limited and low-quality research. There is no clear evidence that one form of feedback is more effective than another, although it appears that feedback delivered via technology may be as effective as feedback delivered in traditional therapy interventions for children with DCD. Further exploration is required from appropriately powered and well-designed trials.

**Systematic Review Registration:**

https://www.crd.york.ac.uk/prospero/display_record.php?RecordID=175118, identifier (CRD42020175118).

## 1 Introduction

Children with Developmental Coordination Disorder (DCD) typically experience issues with motor performance that restrict participation in activities of daily living, impact academic performance, and increase risk of psycho-social difficulties (1–4). Clumsiness, together with slow and inaccurate gross and fine motor skills, are defining features of DCD. Long-term consequences of DCD include lower self-concept and self-efficacy, reduced physical and social participation, and increased rates of anxiety and depression that extend into adolescence and adulthood (3–8). Without intervention or support, between 50%–70% of individuals with DCD will continue to have motor and subsequent psychosocial difficulties into adolescence and adulthood (9).

It is well established that any form of physical activity and/or intervention is important for motor learning and performance in children with and without motor difficulties (10, 11). Children with DCD have difficulty acquiring, retaining, executing, and transferring fundamental motor skills (9, 12). Importantly, like typically developing (TD) children, research suggests children with DCD can successfully learn new motor skills and tasks, however, task type and difficulty significantly moderate motor performance and learning outcomes beyond their TD peers (13, 14). For example, children with DCD can learn simple motor tasks just as quickly and accurately as their TD peers, however they perform more slowly and less accurately on more complex motor tasks (14). It has been hypothesized this might be due to their reduced ability to use internal and/or external feedback to predict and update their stored motor plan, and that children with DCD may benefit from amplified feedback mechanisms to facilitate the motor learning process.

**Figure 1:**
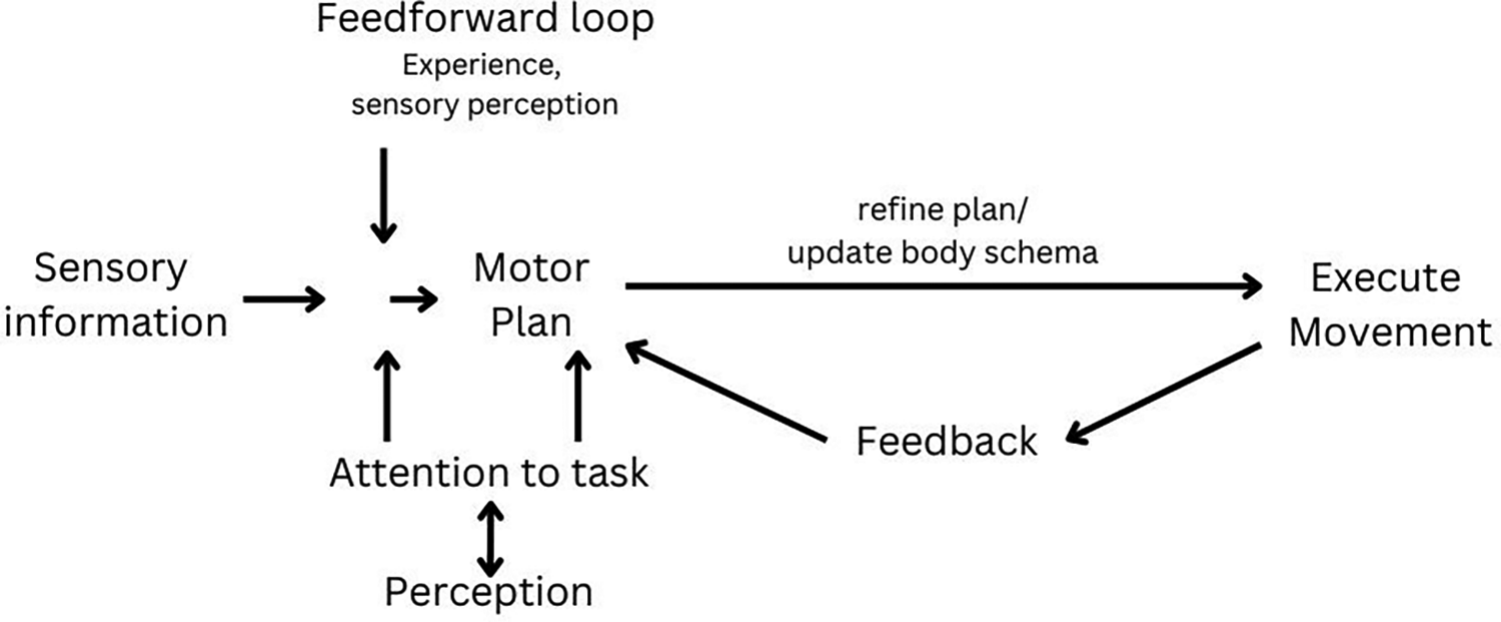
Traditional motor loop.

To produce movement, an individual must analyse the environment and generate movement to meet the demands of the task. This is typically facilitated via feedforward and feedback control processes (15). Before movement execution, sensory information of our surroundings and prior experience of the movement are used to create a predicted motor plan of the desired movement (feedforward control). As a movement is executed, sensory feedback is analysed during [knowledge of performance (KOP)] and after [knowledge of results (KOR)] the movement and compared to the desired motor plan (feedback control). Mismatches between the predicted and executed movement then result in modification and updating of the motor plan that occurs in real time. The updated and stored motor plan is then available for the prediction and planning of future movement (15) (Figure 1).

Feedback is both a fundamental and crucial element to the motor control process (16–19). In motor learning, feedback can be defined as movement related information that is “fed-back” to the learner and can be provided before, during (performance) or after (results) a movement. Feedback information can be provided intrinsically by specific body movements and sensation of movement within the learner's body (internal feedback), or extrinsically from different environmental cues and task relevant cues from outside the learner's body, such as via visual, auditory, or tactile feedback (external feedback). Common methods of delivering feedback can be through guided discovery and instruction (KOR and KOP), providing internal and external cues by directing the learners focus of attention (FOA), and by manipulating constraints of the task or environment. Each method can be delivered at any point in the motor control process (20).

Augmented feedback is also of particular interest in motor learning and performance as it can provide an individual with amplified internal and/or external knowledge of an action over and above the natural information given in the environment (demanding higher attention). Numerous studies have investigated the effect of different forms of augmented feedback, including feedback delivered by visual, auditory, and tactile modalities, in clinical and non-clinical adults and children (16, 21–23). Research outcomes suggest augmented feedback that is delivered using various methods, and at different frequencies and quantities, both have advantages depending on the population group and skill being learnt (23–26).

**Figure 2:**
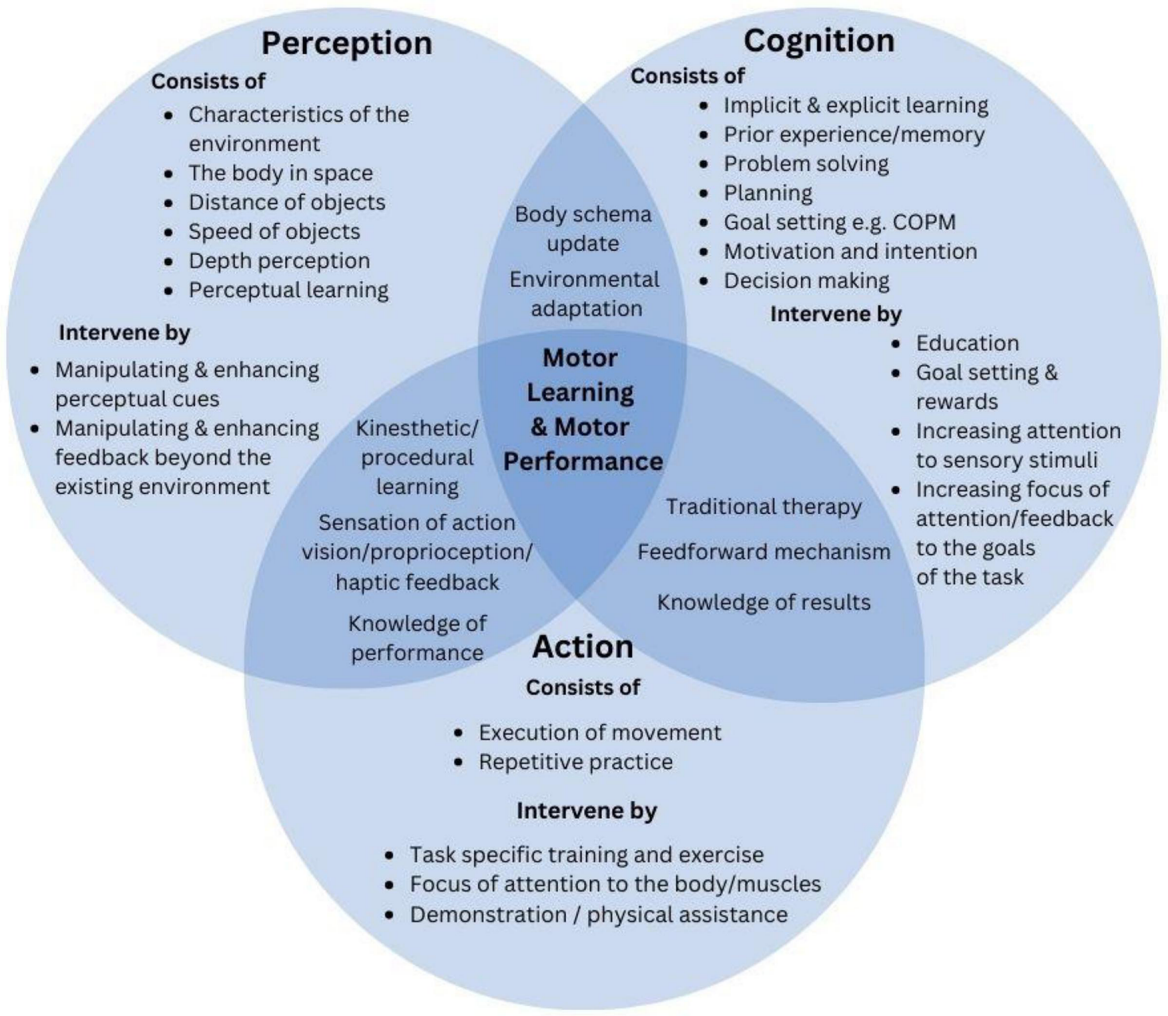
Demonstration of how augmented feedback may be used to manipulate change and /or enhance feedback beyond traditional training methods. This figure combines therapeutic, neuroscience and motor learning theory to display the unidirectional nation of the use of feedback for motor skill training, using the perception, cognitive, and action model.

Traditional therapy and training is typically directed by the interaction of one or more of the three systems in motor control—action, perception, and cognition (PCA)—to promote motor learning. Augmented feedback may be particularly useful to amplify PCA demands of the task. Figure 2 demonstrates where augmented feedback may be manipulated, changed and/or enhanced beyond the traditional therapy models and natural environment to facilitate the learner's ability to recognise and update their motor plan more effectively and refine their motor control loop (27, 28). For example, using the Canadian Occupational Performance Model where goal setting, planning and decision making are a large focus of motor performance, is largely centered within the **Cognitive** domain of performance (see Figure 2) (29). Incorporating ideas of augmenting feedback in this area could include enhancing KOR by encouraging the implicit explanation and/or exploration of the results of the action through guided discovery and instruction, or by tacitly facilitating memory and experience to support motor learning.

Training can also be directed in the **Action** phase where the execution of movement and repetitive practice can increase exposure to a motor skill (for example task-specific training). It may be possible to incorporate or enhance augmented feedback in the action phase by promoting KOP and FOA to kinaesthetic cues during movement.

Within the **Perception** domain, the use of perceptual cues from the learner's environment can also assist the learner—potentially by updating their body schema to support their feedforward learning and therefore their motor plan. Interventions here can also incorporate training via attending to specific sensory stimuli by highlighting one sense over another, by directing one's FOA to their body within their environment, or by manipulating environmental constraints. As demonstrated in Figure 2, it is important to recognise that these systems or domains do not work in isolation, nor are they mutually exclusive.

In addition to traditional training methods, technology appears to be a promising way to deliver augmented feedback at all stages of the motor control loop due to its usability, adaptability, and popularity. Technology in clinical practice has become a popular option for play and rehabilitation for children with DCD (9, 30–32), however, research is limited. Technology can be used to enhance the feedback the learner receives (visual, auditory, haptic) in real time, both during (concurrent or KOP) and after (terminal or KOR) the movement. Providing real-time concurrent feedback may further assist intrinsic and extrinsic feedback mechanisms, that may ultimately help to facilitate successful motor learning through the motor control loop. Promisingly, positive outcomes from augmented feedback using technology have been found in children and adults with low motor abilities, for example cerebral palsy and brain injury (33–38). However, it remains unclear whether augmented feedback using technology has the same motor skill benefits for children with DCD as other clinical populations.

While feedback is important for learning, it remains unclear if any form of feedback is superior to another for motor learning and/or motor performance for children with DCD. Therefore, this systematic review aimed to investigate the effectiveness of different types of feedback, compared to other forms of feedback, or no additional feedback, on motor learning and motor performance outcomes in children with DCD. Findings may lead to a better understanding of the influence of feedback on motor learning and motor performance and could inform the best feedback modalities for future research and clinical interventions for the DCD population.

## 2 Methods

### 2.1 Protocol and registration

The systematic review protocol was registered with the PROSPERO international database and was accepted on 5th July 2020 (registration number CRD42020175118).

### 2.2 Search strategy

The Preferred Reporting Items for Systematic Reviews and Meta-Analyses (PRISMA) guidelines were used to structure the review (39). The following databases were searched on 24th January 2024: MEDLINE, Web of Science, EMBASE, Cochrane, PEDro and OTseeker. The search strategy was developed and trialled in MEDLINE and was adapted to be used in all included electronic databases. The search strategy is available in Supplementary document 1. Two researchers (EW and JB) independently reviewed titles, abstracts, and full texts of the searches to find included studies. Conflicts were reviewed by the two researchers and if a decision could not be reached, a third independent reviewer was consulted (SH). Reference lists of included studies were pearled to identify any missing studies that were not identified in the initial search that met the inclusion criteria.

### 2.3 Inclusion criteria

This review employed the PICOS criteria (population, intervention, comparator, outcome, and study design) to inform the inclusion criteria. Studies had to meet the following criteria: (1) children aged 5–12 years with probable or diagnosed DCD (intervention and control groups), as indicated by a score ≤ 16% percentile on the MABC-2 or low percentile quadrant on other motor performance assessments (e.g., DCD-Q), (2) participants needed to receive a motor skill intervention that authors state the use of one of more forms of feedback, (3) a control group was required who were either receiving an active intervention, that was either a different form of feedback or a traditional training method without feedback, and, (4) included trials needed to include a motor learning and/or performance outcome measure. No restrictions were placed on study design. Conference abstracts and protocols were excluded. Included studies were limited to primary peer-reviewed studies, with no grey literature included.

This review included studies that used and reported on any form of feedback, including but not limited to, internal and/or external feedback, FOA (internal or external), KOR, KOP, or augmented feedback. Interventions that did not specify the use of a specific form of feedback were excluded, as feedback was not isolated, and therefore, it would not be possible to distinguish the effect of feedback as opposed to repeated practice. Motor learning was defined as the ability to acquire, retain, and execute a motor task, due to practice of a particular motor skill. Motor performance was defined as the ability to perform a motor task. The categorisation of articles either assessing motor learning or motor performance was determined by what the authors reported on assessing. For full inclusion and exclusion criteria, see Table 1.

**Table 1:** Inclusion and exclusion criteria.

Inclusion	Exclusion
Study types—primary peer reviewed studies (e.g., RCTs, randomised cross-over, non-randomised trials, cross sectional, controlled trials, case control etc.). Children aged 5–12 years with probable or diagnosed DCD (intervention and control groups), as indicated by a score ≤ 16% percentile on the MABC-2 or low percentile quadrant on other motor performance assessments (e.g., DCD-Q). Receiving a motor skill intervention using one or more forms of feedback for children with DCD, including those delivered via technology (e.g., intrinsic, extrinsic, knowledge of performance, knowledge of results etc.). Required to have a control group receiving an active intervention, either comparing different forms of feedback or a type of feedback versus traditional (no feedback) training. Use of a motor skill outcome tool/assessment	Study types—conference abstracts, protocols, systematic reviews, reviews, and meta-analyses. Children diagnosed or identified as having co-morbidities of other neurological or physical disorders (e.g., autism spectrum disorder, cerebral palsy). Studies using a control group of typically developing children only. Control groups that received no intervention or were required to continue usual activities only.

DCD, development coordination disorder; DCD-Q, developmental coordination disorder questionnaire; MABC-2, movement assessment battery for children, 2nd edition.

Due to the increase in popularity of technology in therapy, interventions delivered via technology in comparison to a traditional training group were included due to the technologies' stated visual, auditory, and/or haptic feedback properties. A technology intervention was defined to be one that used a gaming console device to deliver motor skill practice as the therapeutic intervention, for example using the Nintendo Wii Console. Games that did not have a motor skill focus e.g., a focus on cognition such as Brain training for Nintendo Switch, were not included.

### 2.4 Data extraction and synthesis

Data from all included studies were extracted into a customized Microsoft Excel spreadsheet by one researcher (EW) and a second reviewer (JB) randomly selected and independently reviewed 50% of included studies to ensure no errors were made. Data that were extracted included: study design, participant characteristics (including sample size, mean age, male:female ratio, diagnosis criteria), intervention type (including type of feedback, duration and frequency, adherence to protocol), comparators, outcomes assessed (motor skill outcome) and results. There was not sufficient homogeneity within the included studies (interventions, outcomes, or feedback type) to conduct a meta-analysis, hence, data were synthesised narratively.

### 2.5 Quality assessment

The Cochrane Collaboration's revised tool was used to assess risk of bias in randomized controlled trials, with additional considerations for crossover trials (40). Additionally, the Cochrane risk of bias in non-randomized studies—of interventions (ROBINS-I)—was used to critically appraise the remaining non-randomized included studies (41). For each tool, studies were rated as having high, low, or unclear risk of bias in each of the relevant categories. Two researchers (EW, JB) critically appraised each included study.

## 3 Results

### 3.1 Study selection

**Figure 3:**
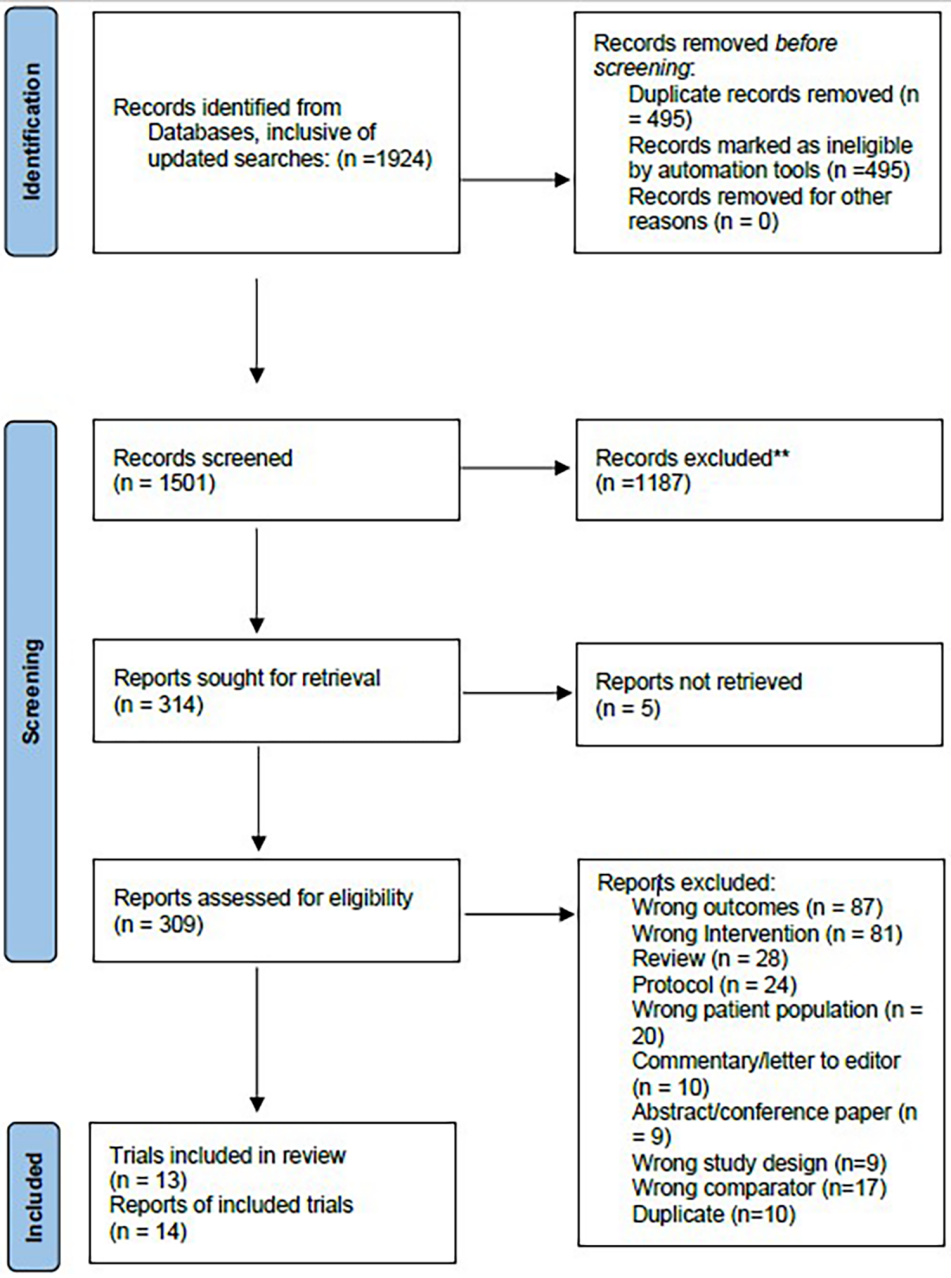
PRISMA flow diagram of included studies.

The database search yielded a total of 1,924 results. Four hundred and ninety-five duplicates were removed. After title and abstract screening, 309 full texts were retrieved and independently reviewed by two researchers. A total of 14 published articles from 13 unique trials were included in this review (Figure 3). One trial reported different outcomes in two separate papers, therefore, both articles were included in this review (42, 43).

### 3.2 Study characteristics

Six RCTs, represented in seven articles (42–48), four randomized crossover design trials (49–52) and three quasi-experimental designs (31, 53, 54) were included in the systematic review. Eight trials assessed internal FOA compared to external FOA, one trial assessed KOR and the remaining four trials were included due to providing augmented feedback delivered via technology in the intervention group, which was tested against traditional therapy.

Data from a combined 392 children with DCD (mean age 5–12 years, mean individual study sample size 5–91) were included. Two articles did not provide the mean age of participants (44, 51), however, ages of participants were restricted by inclusion criteria to between seven and twelve years of age. Six trials used a version of the diagnostic and statistical manual of mental disorders (DSM), relevant to the year the studies were conducted, to confirm a diagnosis of DCD. Two trials used the DSM-IV (31, 44) and four trials used the DSM-V (42, 43, 52–54). The remaining seven trials did not state the use of a diagnostic manual for the confirmation of a DCD diagnosis (45–51). Eleven trials used the MABC-2 assessment to diagnose a motor impairment (DSM-V criterion A), where participants were included if they scored ≤16th-20th percentile (31, 42–49, 52–54). The remaining two trials used other measures to assess criteria for DCD (50, 51). Five trials did not exclude participants based on an indication of ADHD or hyperactivity (31, 46, 50, 53, 54).

While all included articles reported motor learning and/or performance outcomes pre and post intervention, only four articles conducted follow up assessments post intervention, at 48 h, 6–8 weeks and 3 months respectively (46–48, 53). Three of the fourteen articles reported on levels of participant adherence to the prescribed interventions (31, 42, 45) with all reporting adherence of above 80%.

Six of the included trials provided an intervention that was comparable to a traditional therapeutic intervention directed by a therapist (31, 42, 43, 46, 48, 50, 53). The remaining seven trials completed interventions that followed a strict protocol to perform a motor skill (44, 45, 47, 49, 51, 52, 54). Intervention dosage and frequency of included multi-session trials, differed significantly, ranging from 10 to 60 min in duration, over 3–12 weeks for controlled trials. Table 2 lists the characteristics of the individual studies.

**Table 2:** Study characteristics.

Author, year, country, study design	Sample characteristics			Intervention			
	DCD participants	Mean age (SD), male: female	Diagnosis of DCD	Intervention	Experimental groups	Frequency of feedback	Intervention duration, adherence
Traditional feedback methods							
Jarus et al. (44), Canada RCT	*n* = 12 Internal feedback group: *n* = 5 External feedback group: *n* = 7	8–12 years no gender information	DMS-IV; criteria A, B, C, D, MABC-2, ≤5% Co-morbidity screen: Yes	Computer tracking task	IFOA: instructed to focus on movements of their upper limb EFOA: instructed to focus on the computer screen and movements of the joystick	Feedback provided every 5/50 trials during the acquisition phase	3 weeks 4 × 1-hour sessions (Day 1–3: acquisition, day 4: retention & transfer) Adherence: not reported
Khatab et al. (51), Iran Within participants randomised cross over design	*n* = 20	7–11 years, 20:0	Motor observation questionnaire for teachers (Persian), ≤ 16th percentile Co-morbidity screen: No	Dart throwing performance	IFOA: related to the movement of the arm and fingers EFOA: related to the target	Feedback provided at the beginning of each trial block (10 throws) ×5	2 consecutive days x1 session per condition (counterbalanced) Adherence: not reported
Li et al. (45), Taiwan RCT	*n* = 91 EF: *n* = 28 IF: *n* = 32 NF: *n* = 31	12.45 (0.28) EF: 12.45 (0.26) IF: 12.40 (0.32) NF: 12.49 (0.26) Gender ratio unclear	DSM not stated, criteria for inclusion met: A & D MABC-2; ≤5% Co-morbidity screen: Yes	Pole holding task and postural stability	IFOA: focus attention on their hands EFOA: focus on the midpoint of the pole NFOA: no specific instruction	Feedback provided at beginning of task (first 5 trials). Followed by a check of focus after each trial (5)	1 session Five 60-s trials 60 s rest between trial intervals Adherence: Participants excluded if used incorrect FOA
Miles et al. (46), UK RCT	*n* = 30 no group data.	9.07 (.87), 19:11	DSM: not stated Diagnosed by an OT MABC-2, ≤5% Co-morbidity screen: Yes	Catching and throwing task. Instructional video with FOA (QET group or TT group), followed by a training session	IFOA: technical training, standard feedback direction about body position/action EFOA: QET, enhanced feedback through visual gaze direction	Visual feedback provided at beginning. Verbal feedback provided after every 5/60 trials. Then completed 25 attempts no feedback. No feedback at retention.	×2 sessions 1 × training 1 × retention (6–8 weeks later) Adherence: not reported (feedback)
Noordstar et al. (53), Netherlands Quasi-experimental study	*n* = 31 KOR: *n* = 20 Usual: *n* = 11	Intervention 8.15 (0.93), 13:7 Usual: 8.09 (1.14), 8:3	DSM-V, criteria A, B, C, D MABC-2, ≤16% Co-morbidity screen: no	Perceived competence provided by positive, specific, and progress feedback to enhance self-perceptions	KOR group Treatment as usual group	Set specific goals Provided KOR feedback in every session	12 weeks 1 × 30-min session, p/week Adherence: not reported Therapist lead intervention
Norouzi Seyed Hosseini et al. (47), Iran RCT	*N* = 20	8.45 (1.67) 20:0	MABC-2 ≤ 20%	Bimanual wrist coordination accuracy	IFOA: traditional training, no additional feedback EFOA: QET, enhanced feedback through gaze direction	Video shown at beginning of session. Frequency and intervals not reported.	4 weeks 2 × 40-min group sessions p/week Adherence: not reported
Psotta et al. (52), Czech Republic Within participants crossover design	*n* = 18	10.1 (0.6)	DSM-V, Criteria: A, B, C, D MABC-2, ≤15% Co-morbidity screen: yes	Countermovement vertical jumps	IFOA: focus on the swing of your arms EFOA: focus on getting as close to the ceiling as possible NFOA: no instruction	Focus provided before each trial (3) for each condition	×1 session 3 conditions (counterbalanced) 1 min interval between conditions Adherence: not reported
Van Cappellen -van Maldegem et al. (54), Netherlands Quasi-experimental pre-post-test design	*n* = 25 IFA: *n* = 12 EFA: *n* = 13	6.92 (1.7), 23: 3	DSM-V, Criteria: A, B, C, D MABC-2, ≤16% Co-morbidity screen: No	“Slinger ball” throwing task	IFOA: focus on body movement EFOA: focus on external environment/ ball	Feedback delivered via a schedule during practice trials No feedback provided at post-test	x3 sessions across 3 weeks Adherence: not reported Therapist directed intervention, feedback schedule
Wood et al. (48), United Kingdom RCT	*n* = 21 QET: *n* = 11 TT: *n* = 10	8.6 (0.94), 15:6	DSM: not stated MABC-2, ≤5% Co-morbidity screen: Yes	Catching and throwing task.	IFOA: Standard feedback direction about body position/action EFOA: QET, enhanced feedback through gaze direction	Instructional video, reinforced instructions, researcher discretion on feedback frequency during task	4 weeks 1 × 1hr session p/week Adherence: not reported
Augmented feedback via technology							
Bartov et al. (49), Israel Cross-sectional study, counter-balanced	*n* = 27	9.02 (1.18) 17:10	MABC-2 ≤ 15%, DCD-Q ≤ 57 Co-morbidity screen: no	Handwriting on a tablet	Without visual feedback. Black writing With visual feedback. Colour of the writing changed with applied pressure Red: increased pressure Black: moderate pressure Blue: low pressure	Immediate visual feedback according to the degree of pressure provided	8 weeks 1 × 20-min writing session, p/week (performed both conditions, with and without feedback, in each 20-minute session) Adherence: not reported
Cavalcante Neto et al. (42), Brazil RCT	*n* = 32 Wii: *n* = 16 TST: *n* = 16	8.28 (0.81), 24:8	DSM-V, Criteria: A, B, C, D MABC-2, ≤16% Co-morbidity screen: yes	Wii sport: 6 selected games to represent 6 motor skills TST: 6 motor skill matched activities		Feedback provided via each platform and therapist directed feedback during sessions	Wii & TST: 8 weeks 2 × 60-min sessions p/week Adherence: minimum of 80% attendance Therapist lead intervention
Cavalcante Neto et al. (43), Brazil RCT	*n* = 32 Wii: *n* = 16 TST: *n* = 16	8.28 (0.81), 24:8	DSM-V, Criteria: A, B, C, D MABC-2, ≤16% Co-morbidity screen: yes	Wii sport: 6 selected games to represent 6 motor skills TST: 6 motor skill matched activities		Feedback provided via each platform and therapist directed feedback during sessions	Wii & TST 6 weeks 2 × 60-min sessions p/week Adherence: not reported Therapist lead intervention
Ferguson et al. (31), South Africa Quasi-experimental design	*n* = 46 Wii: *n* = 19 NTT: *n* = 27	7.93 (1.21), 24:22	DSM-IV, Criteria: A, B, C, D MABC-2, ≤16% Co-morbidity screen: No	Wii fit training: 18 available games, children instructed to play one game twice before moving on NTT: outdoor games with others		Wii: Encourage and motivation to continue the intervention NNT: Provide positive feedback to support learning and goals	Wii; 6 weeks 3 × 30-min group sessions p/week Adherence: 98% NNT; 9 weeks 2 × 45–60 min group sessions p/week Adherence: 96% Therapist lead intervention
Hammond et al. (50), United Kingdom Randomised cross-over, Pilot study	*n* = 18 Wii: *n* = 10 JAP: *n* = 8	9.03 (1.29), 14:4	Bottom quintile on the DCD-Q, or formal diagnosis of DCD BOT-2, ≤31% Co-morbidity screen: No	Wii fit training focused on balance and coordination (total of 9 games) Jump Ahead program (JAP)			Wii; 4 weeks x3 10-minute sessions p/week JAP; 4 weeks 1 × 1 h. group session p/week 2.5 months between crossover of intervention Adherence: not reported Not therapist lead. Supervised by teaching staff

BOT-2, Bruininks-Oseretsky test of motor proficiency edition 2; DCD, developmental coordination disorder; DCD-Q, developmental coordination disorder questionnaire; DSM-IV, diagnostic and statistical manual of mental disorders, 4th edition; DSM-V, diagnostic and statistical manual of mental disorders, 5th edition; EFOA, external focus of attention; FOA, focus of attention; IFOA, Internal focus of attention; JAP, jump ahead program; MABC-2, movement assessment battery, 2nd edition; TST, task specific training; NFOA, no focus of attention; NNT, neuromotor task training.

#### 3.2.1 Quality of included studies: risk of bias

**Figure 4:**
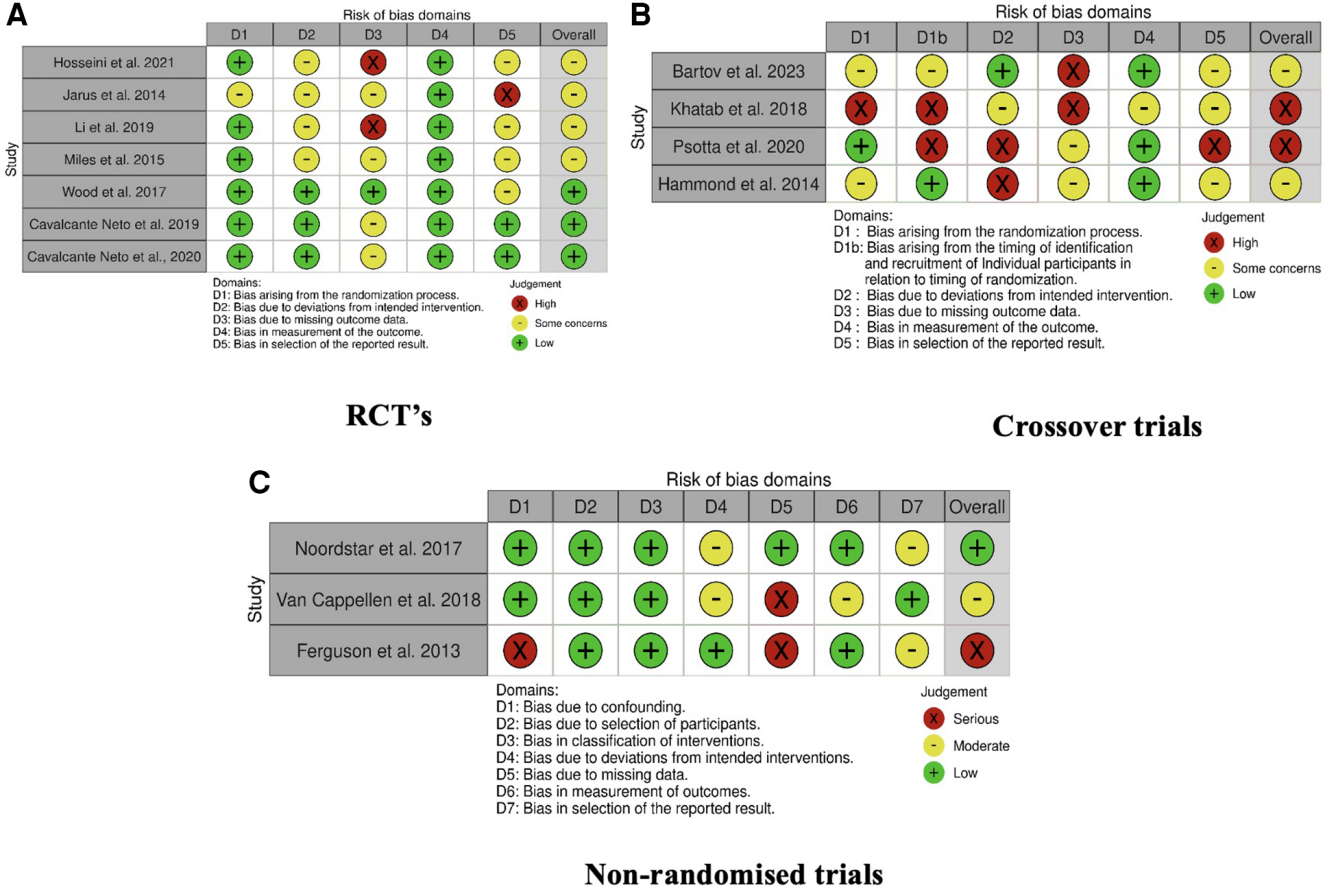
Risk of bias for individual studies. (A) RCT's, (B) crossover trials, (C) non-randomized trials.

The risk of bias assessment is presented in Figure 4. All but three articles (42, 43) had domains that were either unclear or had a high risk of bias. Four of the RCT papers were poorly reported and consequently, the risk of bias was unclear in several domains (44–47). Poor methodology in all four crossover trials resulted in a moderate-high risk of bias (49–52). The non-randomized trials revealed unclear reporting of methods with some concerns about the fidelity of the interventions provided, however, most other domains revealed a low risk of bias (31, 53, 54). Due to poor reporting of methods and results, authors of three included articles were contacted, with no response received.

#### 3.2.2 Intervention duration and intensity of included studies

Four of the eight trials that explored internal FOA and external FOA were single session protocols (45, 46, 51, 52), with only one trial including a follow up retention session (46). The remaining four trial interventions ranged between 4 and 12 weeks, consisting of 4–12 sessions (47–49, 53, 54).

One trial examined the effect of increased KOR during a 12-week therapy program (53). Both groups received one 30-min session per week consisting of the same treatment, however, the intervention group received increased positive and specific feedback on progress towards achieving participant goals throughout the treatment sessions.

The five trials that investigated feedback delivered by technology varied in intervention duration for the technology groups and traditional training groups. Cavalcante Neto et al. (42, 43) matched group duration and frequency, consisting of 2 × 60-min sessions per week for up to 8 weeks. Bartov et al. (49) also matched group duration and frequency, each group completing eight 20-minute sessions per week for 8 weeks. Ferguson et al. (31) reported the Wii intervention group participated in 3 × 30-min sessions per week for 6 weeks, compared to the neuromotor task training group who completed 2 × 45–60-min sessions per week over 9 weeks. Lastly, Hammond et al. (50) reported the Wii group participated in 3 × 10-min sessions over 4 weeks, compared to the Jump Ahead Program group who participated in 1 × 60-min sessions per week, for 4 weeks. Table 3 lists the outcome measures and conclusions of each included article.

**Table 3:** Outcomes of included studies.

Author, year, country, study design	Outcome measures				Author conclusions
	Pre-post measure	Follow up Y/N	Significant difference, *p*-value if available	Motor learning/motor performance	
Traditional feedback methods					
Jarus et al. (44), Canada RCT	Root-mean-square-measure (RMSE) of the difference between the target path and path traced by the participant	N	No raw data available for FOA	Motor learning under implicit learning conditions	No significant difference was found between internal and external FOA during acquisition, retention, and transfer phases of learning for children with DCD. Authors reported that during acquisition, an external FOA was slightly more beneficial, although not significant. (no statistics were provided from authors).
Khatab et al. (51), Iran Within participants cross over design	Accuracy: Average score of each trial block	N	Main effect of attention: *p* = 0.749 Main effect of trial block: *p* = 0.001	Motor performance	No significant difference was found between an internal or external FOA on dart throwing performance in children with DCD. The main effect of the trial period was significant, meaning all children improved in their throwing scores over the trial period no matter what group they were allocated too.
Li et al. (45), Taiwan RCT	Pole movements: pole sway pole rotation Centre of pressure (COP): postural stability	N	Group × FOA Displacement: *p* = 0.975 Rotation: *p* = 0.863 COP: *p* = 0.727	Motor performance	No significant effect for group × FOA for AP displacement, pole rotation or postural sway. 70.97% of DCD participants in the NF group, reported adopting an internal FOA.
Miles et al. (46), UK RCT	Successful catching performance (average of catching scores) & Qualitative catching score at baseline (BL) to immediate retention (R1) and delayed retention 6–8 weeks post training (R2).	Y Immediate retention 6–8 weeks retention	Catching performance: Main effect for test: BL—R1: *p* < .001 No significant main effect of group Qualitative catching score: trend for significance in QET group at R2 (*p* = 0.068) compared to TT group.	Motor learning & Motor performance	Both groups improved between baseline and immediate retention (BL—R1) in catching performance. No significant difference was detected at follow up (R1—R2), suggesting maintenance of skill acquisition for both groups. There was also no significant difference between groups for the qualitative catching score, however, a trend was revealed for near significance in the QET group at delayed retention.
Noordstar et al. (53), Netherlands Quasi-experimental study (non-randomised)	MABC-2: Pre (T1), post (T2), follow up (T3) DCD-Q: Pre (T1), post (T2), follow up (T3)	Y 3 months	Pre post MABC-2: Main effect of group for performance improvement, (*p* = 0.005) DCD-Q: Intervention × time, (*p* = < 0.001) 3 months MABC-2: maintained for both groups (*p* = 0.003) DCD-Q: maintained for both groups (*p* < 0.001)	Motor performance	There was no significant effect of type of feedback (enhanced KOR vs. usual treatment) delivered between intervention groups for motor performance at T1-T2 or T2-T3, as assessed by the MABC-2, or on improvement of motor difficulties, as reported by parents on the DCD-Q. However, both groups improved in their motor performance and motor difficulties decreased over time after 12 sessions. This was maintained a follow up.
Norouzi Seyed Hosseini et al. (47), Iran RCT	Bimanual wrist coordination accuracy	Y 48 h	Pre-post-test: Group × time: *p = 0.001* Retention: Post test-retention: *p = 0.04*	Motor performance	Both groups improved in bimanual wrist coordination accuracy through participating in the intervention. Participants in the QET group performed more accurately from pre to post test and in the retention phase than the traditional training group, suggesting that visual feedback was more essential for successful performance of a bimanual coordination task.
Psotta et al. (52), Czech Republic Within participants crossover design	Vertical jump performance	N	Attentional focus and group: *p* = 0.549 DCD: IFA vs. EFOA *p* = 0.063 EFA vs. control *p* = 0.394 IFA vs. control *p* = 0.635	Motor performance	No significant difference was found between EFA, IFA and control conditions within the DCD population. A trend was revealed for the DCD group, where an EFOA compared to IFOA led to a slightly higher jump performance.
Van Cappellen -van Maldegem et al. (54), Netherlands Quasi-experimental pre-post-test design (non-randomised)	Throwing accuracy	N	Significant main effect of test, pre-post-test: *p* = 0.024 Interaction of test and focus: no significant difference (no statistical value available)	Motor performance	There was a significant main effect of test from pre to post test for both IFOA and EFOA groups, indicating all children improved in motor performance.
Wood et al. (48), UK RCT	Pre-post-retention Catching performance (total no. balls caught out of 50) Qualitative catching performance score	Y 1 week retention 6 weeks delayed retention	Pre-post-retention Main effect for test: *p* = <0.001 Delayed retention: *p* = 0.351 Qualitative catching scores: Between group differences: *p* = 0.236 Between group differences at delayed retention: QET group, *p* = 0.032 TT group, *p* = 0.529	Motor learning & Motor performance	No significant difference between groups on catching performance scores. Both groups caught significantly more balls from baseline to retention and maintained this improvement at delayed retention. Qualitative catching scores revealed the QET group were observed to significantly improved their catching technique on the qualitative catching score at delayed retention than the TT group who did not significantly improve.
Augmented feedback via technology					
Bartov et al. (49), Israel Cross-sectional study	Acquisition, retention and transfer tests of timing, spatial and pressure variables of handwriting between conditions	N	Timing Time × condition: p = 0.001* Main effect of time: *p = 0.001* Spatial Main effect of time: *p = 0.008* Pressure No main effect. Post-intervention between conditions: *p* = ≤*0.001*	Motor learning	Time: Total time writing decreased overall with intervention, with the feedback group decreasing their total writing time more than the no feedback group. *However, it is noted that pre-intervention writing time was higher for the feedback condition. Overall, no difference was found between conditions at post-intervention. Spatial: There was a main effect for space of letters for both conditions. There was no significant effect between conditions over the intervention period. Pressure: No main effect of pressure. Significant difference was found between the with and without feedback conditions for writing pressure. The writing pressure was increased over the intervention period for the visual feedback condition compared to the no feedback condition. Additionally, both conditions had fewer fluctuations in pressure at post-intervention. Transferability: Authors report an observed ability to transfer the acquisition of writing components to a similar but new task. No statistical difference was measured between feedback groups.
Cavalcante Neto et al. (42), Brazil RCT	MABC-2	N	MABC-2: Between groups: *p* = 0.47 Pre-post change: Wii group: *p* = <0.01 TST group: *p* = <0.01 Balance component: Wii group: *p* = < 0.001 TST group: *p* = <0.001	Motor performance	No significant difference was found between groups for MABC-2 total standard score. However, a significant pre-post change in total motor performance scores on the MABC-2 was found for both groups. The pre-post scores for the balance component of the MABC-2 was significant in both groups.
Cavalcante Neto et al. (43), Brazil RCT	Mean game scores for each task in three phases: phase 1 (session 1–4), phase 2 (sessions 5–8), and phase 3 (sessions 9–12)	N	Main effect of group: No significant differences (no statistical value available) Percentage of change between groups: Table tennis: phase 1–2: *p* = 0.15, phase 2–3: *p* = 0.86 Archery: phase 1–2: *p* = 0.15 phase 2–3: *p* = 0.21 Frisbee: phase 1–2: *p* = 0.02 phase 2–3: *p* = 0.40 Marble balance/balance disk: phase 1–2: *p* = 0.40 phase 2–3: *p* = 0.14 Tightrope walking/balance beam: phase 1–2: *p* = 0.30 phase 2–3: *p* = 0.47 Bowling: phase 1–2: *p* = 0.01 phase 2–3: *p* = 0.82	Motor learning	Game scores were used to assess motor learning in three different consecutive phases. Neither Wii nor TST groups were better than the other for motor learning. Each group significantly improved by participating in intervention. Highest percentage of change was recorded in frisbee and bowling tasks in favour of Wii-intervention, suggesting tasks may be tailored to children in different learning phases with preference of Wii and TST at different stages.
Ferguson et al. (31), South Africa Quasi-experimental design (non-randomised)	MABC-2 (Total test score)	N	Main effect of time × group: *p* = <0.001 Time × group of TSS: Wii, *p* = 0.26 (*d *= −0.50) NTT, *p* = <0.01 (*d *= −4.32)	Motor performance	No significant difference between the Wii fit training and NTT groups were found. Both groups revealed a significant improvement in motor performance over the intervention period. The total test score of the NTT group was significant over the intervention period, whereas the Wii fit training group was not significant, however showed a moderate effect size. NTT training was demonstrated to be superior to Wii training.
Hammond et al. (50), UK Randomised cross-over, Pilot study	BOT-2	N	Main effect, Group × time; *p* = <0.02 No significant main effects of either group (*p* = 0.987) or time (*p* = 0.082)	Motor performance	No significant difference between groups for the Wii fit training vs. the jump ahead program. Improvement in both groups was evident following participation in intervention. Wii fit training may be beneficial to improve motor outcomes in children with DCD.

BOT-2, Bruininks-Oseretsky test of motor proficiency edition 2; DCD, developmental coordination disorder; DCD-Q, developmental coordination disorder questionnaire; EFOA, external focus of attention; FOA, focus of attention; IFOA, internal focus of attention; KOR, knowledge of results; MABC-2, movement assessment battery, 2nd edition; ns, non-significant (value not provided in article); TST, task specific training; NFOA, no focus of attention; NNT, neuromotor task training; QET, quiet eye training.

#### 3.2.3 Types of feedback

Eight trials assessed internal FOA compared to external FOA for motor learning and performance (44–48, 51, 52, 54). One trial examined the effect of increased KOR during a 12-week therapy program (53).

Five trials, represented in four articles, investigated the effect of augmented feedback delivered via technology compared to traditional training methods (31, 42, 43, 49, 50). Three trials used the Nintendo Wii to augment feedback and one used an iPad to deliver immediate visual feedback (49). Control participants received a no feedback handwriting task (49), task specific training (42, 43), neuromotor task training (31) and the Jump Ahead Program (50).

No other articles were found by this review that investigated other forms of feedback for motor learning or performance for children with DCD.

### 3.3 Impact of feedback on motor performance and motor learning

#### 3.3.1 Motor performance

Eleven articles reported on the impact of different forms of feedback on motor performance. Seven of these articles investigated an internal FOA compared to an external FOA (45–48, 51, 52, 54). Only two of these trials including a “no specific FOA” feedback groups on motor performance (45, 52). Six of these seven articles did not find a significant difference between an internal FOA, external FOA or no FOA on motor performance outcomes, including the single session trials (45, 52), multi session trials (46, 48, 51, 54), and trials that included follow up measures (46, 48). The remaining article found a significant difference between visual gaze direction feedback and no gaze direction for a bimanual wrist coordination task (*p = 0.001*) (47).

The two single session trials (45, 52) were the only two trials to include a no feedback group, and both reported no difference between internal FOA, external FOA or no direction feedback groups. Interestingly, Li et al. (45) revealed 70.97% of participants in the no direction group, reported adopting internal FOA feedback principles. Although no significant results were found, it may be important to note that Psotta et al. (52), found a trend for benefits of external FOA on jump performance for children with DCD (*p *= 0.063) compared to internal FOA and no direction groups. Finally, it is noteworthy that the six multi-session articles did not include a “no feedback” group, and that all articles reported improvements in motor performance from baseline (45, 48, 51–54).

Noordstar et al. (53) investigated the effect of using increased specific KOR feedback compared to usual care on motor learning outcomes. There was no significant difference reported between groups for motor performance outcomes at the end of the 12-week intervention period. However, it was reported that all participants, regardless of group, improved in their motor performance due to participation in the intervention (*p = 0.005*), and that this was maintained at a 3-month follow up (*p = 0.003*).

The remaining three articles investigated the effect of augmented feedback, delivered via technology, on motor performance (31, 42, 50). Ferguson et al. (31) reported an overall improvement in motor performance for both groups (*p = *<0.01). Cavalcante Neto et al. (42) revealed a significant pre-post change for the total standard score (*p *= <0.01) and for the balance component score (*p = *<0.001) on the MABC-2 assessment for both groups. Hammond et al. (50) also revealed a significant time effect for motor performance in both groups (*p = *<0.02). Altogether, no significant differences were reported between intervention methods, but again, all three articles revealed overall significant improvements from baseline.

#### 3.3.2 Motor learning

Three articles reported on internal FOA and external FOA for motor learning (44, 46, 48). Additionally, two articles reported on augmented feedback delivered via technology for motor learning (43). All five articles were included as assessing motor learning if the authors stated motor learning as the outcome. All five articles completed outcome assessments for skill acquisition and retention. Interestingly, only two of the five articles reported using skill acquisition, retention, and transfer tests to determine motor learning effects (44, 49).

All three articles that investigated internal FOA compared to external FOA showed that there was no difference between the two methods for motor learning outcomes (44, 46, 48). Two articles that included a follow up assessment reported significant results for maintenance of motor skills on standardized quantitative outcome measures for both groups, suggesting acquisition and maintenance of the learned motor skill despite type of feedback modality (46, 48). Wood et al. (48), revealed a significant improvement in qualitative catching scores for the quiet eye training (QET) group at follow up (*p = *0.032), revealing the QET group (external FOA) were subjectively observed to have significantly improved their catching technique on delayed retention, whereas the traditional training group (internal FOA) had not. Additionally, Miles et al. (46), who also used the QET training method, revealed a trend for significance for the external FOA group for qualitative catching scores at delayed retention (*p = 0.068)*.

Bartov et al. (49) revealed a significant time × condition interaction (*p* = 0.001) in favour of the immediate visual feedback group with handwriting, however, we note that there was a significant difference at baseline between the feedback and no feedback group, which when corrected for, revealed no significant differences between groups. The remaining article investigated the effect of the Nintendo Wii compared to task specific training for motor learning in children with DCD (43). No significant difference between groups for motor learning was reported. However, the authors stated the Wii training group showed the highest percentage change in phase 1 to phase 3 for frisbee and bowling, compared to the task specific training group.

## 4 Discussion

The aim of this systematic review was to investigate the effect of different forms of feedback, compared to other forms of feedback, or no additional feedback, for motor learning and/or performance in children with DCD. Interestingly, eight of the fourteen included articles used either internal or external FOA as feedback during intervention. The remaining articles reported on KOR (one) and intervention delivered via technology (five). This review found that the use of any feedback training strategy (internal FOA, external FOA, KOR, augmented feedback, and no additional feedback), with any intervention (traditional or technology), appeared beneficial to improve motor outcomes in children with DCD. These findings are consistent with previous research that indicates any kind of training is better than none (9, 55). This review also found that augmented feedback delivered by technology may provide equivalent motor skill outcomes as traditional training methods for children with DCD.

### 4.1 Feedback for motor learning and performance

Motor performance appeared to benefit from internal FOA, external FOA, KOR, no additional feedback and augmented feedback modalities (31, 42, 45–48, 50–54). Specifically, visual feedback delivered by QET, appeared to be indicative of a better performance with a specific motor task for children with DCD. This result should be interpreted with caution as only one of three articles using the QET method found a statistical significance between groups (47). Additionally, this review found that no individual article reported statistically significant differences in their sample between internal or external FOA or augmented feedback delivered by technology for motor learning outcomes for children with DCD.

An interesting finding of this review was that only three articles included a “no feedback” group (45, 52). Two of the trials investigated motor performance and were a single session design. Within the trials, participants only completed three and five repetitions of the task and neither trial found a difference between internal FOA, external FOA or no additional feedback groups. The remaining article investigated motor learning and compared external visual feedback to no additional feedback (49). Authors revealed a significant decrease in writing duration, and an improvement with spatial features of the letters for both groups, indicating no difference between additional external feedback and no feedback for motor learning in handwriting. All other multi-session trials did not include a “no feedback” control group and/or included a comparison to a TD group (which was not the focus of this review). From the findings of this review, only one multi-session intervention trial exists where a form of specific feedback is compared to no additional feedback, making it impossible to form a conclusion about the use of a specific form of feedback provided during therapeutic intervention. It appears that all participants who participate in a motor skill intervention, regardless of type of feedback or intervention completed, improved their motor performance outcomes.

Some evidence proposes that children with DCD have increased difficulty with internal feedback information (sensory perception), due to poorer acquisition, retention, and transfer under implicit conditions (unconscious automatic learning), than their traditional developing peers, which may result in less “learning by experience” (20, 44, 56). One hypothesis is that due to the proposed difficulty with implicit learning (44), and potentially limited experience with motor tasks, children with DCD may benefit from a greater focus on an internal approach to encourage the automatic update of feedforward control to enhance motor skill learning outcomes (16). Contrary to this, some researchers suggest that a focus on controlling one's movements (internal feedback) may suppress the automatic update of the motor plan. Therefore it is proposed that an external method to feedback may encourage the implicit learning process by taking the focus away from specific movements of the body, therefore promoting unconscious learning (16, 57, 58). Interestingly, in a single session design, Li et al. (45) revealed that 70.97% of DCD participants in the “no feedback” group reported spontaneously adopting an internal FOA method to assist them to perform the movement, however, no differences were found in this review between internal or external FOA. No other studies that included a “no feedback” group reported on the participants approach to feedback during a task. An external approach to feedback has been shown to be more beneficial for motor learning in adults, however as this review has shown, limited and variable research exists for the DCD population. This review found very limited evidence to detect a difference between forms of feedback for motor learning or performance for children with DCD.

Within traditional therapeutic methods, therapists aim to provide external feedback to supplement naturally occurring task-intrinsic (sensory) information to improve the learner's motor performance, and to consequently encourage increased experience of the skill to improve motor learning. Typically, this is delivered by KOR and KOP. This review only found one article that investigated the effect of enhanced KOR on motor performance for children with DCD (53). No significant improvement with KOR was found, suggesting this additional external feedback, provided during a traditional motor skill program, did not offer benefits for motor performance beyond traditional training for children with DCD (53). This finding should be considered with care due to the limited evidence found and the methods employed in the included trial. Therapists who participated in the usual-care group, although blinded to the true outcomes of the trial, were not guided by standardized protocols, nor were their therapeutic methods collected, meaning it is highly possible additional feedback was also provided in the usual-care group. Additionally, therapists delivering the enhanced KOR were not guided by feedback schedules nor required to report the level of feedback provided. This finding is surprising, as the benefits of KOR and KOP are well documented as being beneficial for motor learning and performance in other populations, however, we did not find sufficient articles that focused on these forms of feedback for the DCD population. Additionally, no trials were found that investigated KOR and/or KOP for motor learning. More research is required, which employs standardized protocols and reporting, to investigate the influence of KOR and/or KOP for motor learning and performance for children with DCD.

Despite the importance of feedback during intervention tasks, none of the included articles reported on feedback frequency and intensity. Most articles reported the use of a feedback schedule in their methods; however, this was not explored further. Existing literature proposes the importance of feedback frequency during intervention to improve motor skills. It has been shown that for TD children, a lower frequency of feedback (33%) was more beneficial for motor skill learning on a simple task, however a higher frequency (100%) of feedback was more beneficial for a complex task (24). In children with CP, a moderate frequency of feedback (50%–62%) has been shown to be more beneficial during tasks, however, this may be largely influenced by task complexity and individual preferences (23). These findings appear consistent with children with DCD, where more feedback and repetitive practice may be required to achieve similar motor skill outcomes as their typically developing peers, and that task type and complexity may significantly alter acquisition and retention of motor skills (13, 14, 25). Additionally, literature suggests self-controlled feedback scheduling may be a good way to facilitate improved motor learning across all age groups, including in children (59, 60). Surprisingly, this review did not find any articles that reported on these forms of feedback scheduling for children with DCD. Articles included in this review did not have adequate reporting of feedback frequency to determine its effect on motor learning or performance for children with DCD and is an important consideration for future trials.

### 4.2 Augmented feedback delivered via technology vs. traditional training

Despite the positive applications and outcomes seen in children and adults with motor impairments from the use of virtual and augmented reality systems, this review only identified four trials, represented in five articles, that investigated the effect of augmented feedback via technology for children with DCD (31, 42, 43, 50). Promisingly, results identified that the use of technology interventions to deliver augmented feedback appear to be equivalent to traditional therapy, suggesting that technology could be beneficial as part of a therapeutic program from children with DCD. Technology may introduce options to train with or without a therapist and/or with greater intensity at home or in the clinic. Additionally, it may provide a platform that individuals can readily identify with and be used as a tool to enhance motivation within therapy. Positive effects on motor function using technology have been evidenced for other clinical and non-clinical populations, such as in cerebral palsy (33). This may support a rationale for the potential benefits for children with DCD who may share similar internal feedback processing challenges.

Current clinical practice recommendations suggest that children with DCD need better ways to self-evaluate their performance (9, 12), and instant or concurrent feedback of a person's movement pattern via technology has been popular due to its ability to provide enhanced multisensory feedback (auditory, visual, haptic). However, evidence is still mixed about the true effects of this style of feedback on motor learning in all populations (61). While results are promising for the use of technology in this review, three of the four trials rated a moderate-high risk of bias (31, 49, 50). These studies were heterogeneous in nature, with mixed methods differing significantly in their motor skills training, duration, and frequency of interventions between the Wii and traditional training intervention groups (31, 50). Only two trials matched the motor skills being learnt between intervention groups (42, 43, 49). Further research needs to be conducted to investigate the influence of augmented feedback on motor learning and performance in all populations and better-quality evidence needs to be conducted for the use of technology in the DCD population.

### 4.3 Limitations

This review was completed using a narrative synthesis due to the low quality and heterogeneity of the included trials, including low sample sizes, low quality reporting of intervention, variability of study designs, interventions and outcome measures used. Diagnostic criteria and motor skill outcome measures for DCD varied between trials. Trials that did not use the DSM-IV or DSM-V to make a diagnosis of DCD or probable DCD were not excluded from this review. Additionally, reporting of co-morbidities was poor, making some data at risk of being confounded and reducing the reliability or validity of outcomes. This may mean that some of the data cannot be generalized to the DCD population alone due to a mixed sample. Included trials varied significantly in motor skills and interventions used, and surprisingly, most articles had low quality of reporting intervention and outcome measures. Trials also varied in intervention frequency, intensity and duration, and the risk of bias was found to be moderate to high for the majority of included studies. Only four of the included trials included a follow up to the intervention, meaning it cannot be concluded whether reported effects remain long term. The potential inclusion of the above-mentioned factors means that we cannot confidently draw a conclusion on the effect of different forms of feedback for motor learning or performance for children with DCD.

### 4.4 Implications and recommendations for future research

This review revealed there is a limited amount of high-quality research investigating the influence of different forms of feedback on motor learning and motor performance for children with DCD. We identified several gaps in the literature that require investigation for the DCD population. Further high-quality research needs to be conducted to investigate the best feedback modality, frequency, and approach options for the DCD population (62). Future studies should be appropriately powered, ensure motor skills being learnt are consistent between the intervention and comparator groups, and include appropriate acquisition, retention, and transfer assessments to determine how different forms of feedback may affect motor learning and performance in children with DCD. A “no feedback” comparator group should also be included to determine the true effects of feedback during intervention across different tasks. Studies also need to ensure they have clear aims and standardized method of delivering different forms of feedback, and at what frequency, within interventions. Future trials also need to include a standard method for classifying DCD, such as using the DSM-V criteria.

## 5 Conclusion

It is well known that children with DCD who engage in therapeutic interventions have significantly better motor performance outcomes than those who do not (10, 55). However, limited evidence exists about the effect of an internal or external approach to feedback during an intervention and its influence on motor learning and performance outcomes. This review identified that both internal and external FOA and KOR during an intervention were beneficial for motor learning and performance outcomes for children with DCD, with no superior effect for one method over the other. It was also found that interventions delivered via the use of technology, which delivered augmented feedback, were equivalent to traditional therapy for motor learning and performance outcomes. It appears that any intervention is better than no intervention, and that technology may be a promising avenue to deliver more independent interventions of therapeutic equivalence to traditional therapy for children with DCD. More research is required to clarify the role of feedback in DCD interventions.

## Data Availability

The raw data supporting the conclusions of this article will be made available by the authors, without undue reservation.
