# Supplementary material for: Evaluating the influence of feedback on motor skill learning and motor performance for children with developmental coordination disorder: a systematic review
Source: Front Pediatr. 2024 Apr 19;12:1327445. doi: 10.3389/fped.2024.1327445 (PMC11066222; doi:10.3389/fped.2024.1327445)
Supplement: Supplementary file 1 [file Datasheet1.pdf]

Supplementary 1

Search Strategy

Example of Medline search:

((Feedback/) OR (Focus of attention/) OR (feedback OR motor feedback OR intrinsic feedback OR extrinsic feedback OR focus of attention) OR (augmented feedback OR knowledge of results OR knowledge of performance OR attention) OR (video game* or game* or gaming or virtual reality or augmented reality or serious game*)) AND ((Motor Skills/) OR (motor skill* OR motor learning OR motor perform* OR motor outcome*) OR (practic* or training or intervention*)) AND ((Child/) OR (child* or kid* or young person*)) AND ((Motor Skills Disorders/) OR (developmental coordination disorder or DCD or minimal brain dysfunction or minor neurological dysfunction* or MND or dyspraxia or perceptual?motor disorder or perceptual?motor dysfunction or specific developmental disorder of motor function or SDDMF or motor learning difficult* or motor learning disorder* or coordination disorder*)).
